# Herba *Cistanche* (Rou Cong-Rong): One of the Best Pharmaceutical Gifts of Traditional Chinese Medicine

**DOI:** 10.3389/fphar.2016.00041

**Published:** 2016-03-01

**Authors:** Zhiming Li, Huinuan Lin, Long Gu, Jingwen Gao, Chi-Meng Tzeng

**Affiliations:** ^1^Translational Medicine Research Center, School of Pharmaceutical Sciences, Xiamen UniversityXiamen, China; ^2^Key Laboratory for Cancer T-Cell Theranostics and Clinical TranslationXiamen, China; ^3^INNOVA Clinics and TRANSLA Health GroupXiamen, China

**Keywords:** *Cistanche* species, Herba *Cistanche*, phenylethanoid glycosides, improvement of brain function, aphrodisiac effect, immune-boosting effect

## Abstract

*Cistanche* species, known as Rou Cong-Rong in Chinese, are an endangered wild species and are mainly distributed in the arid lands and warm deserts of northwestern China. Within Traditional Chinese Medicine (TCM), Herba *Cistanche* is applied as a tonic and/or in a formula for chronic renal disease, impotence, female infertility, morbid leucorrhea, profuse metrorrhagia, and senile constipation. The chemical constituents of Herba *Cistanche* mainly consist of volatile oils, non-volatile phenylethanoid glycosides (PhGs), iridoids, lignans, alditols, oligosaccharides, and polysaccharides. There have been an increasing number of studies focusing on its bio-activities, including antioxidation, neuroprotection, and antiaging. The objective of this review is to introduce this herb to the world. Its taxonomy, distribution, and corresponding biological functions and molecular mechanisms are addressed in this review.

*Cistanche* Hoffmg. Et Link is a genus with in the Orobanchaceae family and includes 22 species throughout the world. The *cistanche* species include the perennial parasite herbs, which commonly attach onto the roots of sand-fixing plants, such as *Haloxylon ammodendron*, *H. persicum*, *Kalidium foliatum*, and *Tamarix* plants (Li et al., 2013b). Generally, *cistanche* species distribute in arid lands and deserts in the northern hemisphere, such as the provinces of Xinjiang, Inner Mongolia, Gansu, Qinghai, and the Ningxia Autonomous Region in China in addition to similar regions of countries such as Iran, India, and Mongolia (Jiang and Tu, 2009). The growth and cultivation of *cistanche* species require severe environmental conditions: extreme arid climate, depauperate soils, large temperature difference, intensive sunshine, and less than 250 mm of annual precipitation (Qiao et al., 2007). Among the 22 species in the world, six are found in China according to the Taxonomical Index of Chinese Higher Plants (Plant Institute of Chinese Academy of Science, 1994); however, a follow-up study indicated that only 4 species and 1 variation of *cistanche* exist in China, including *Cistanche deserticola* Y. C. Ma, *C. tubulosa* (Schenk) R. Wight, *C. salsa* (C. A. Mey.) G. Beck, *C. salsa* var. albiflora P. F. Tu et Z. C. Lou and *C. sinensis* G. Beck (Jiang and Tu, 2009).

The Herba *Cistanche* (Rou Cong-Rong in Chinese) was first recorded in Shen Nong’s Chinese Materia Medica, where it was referred to as the dried succulent stems of the *cistanche* species (Karalliedde and Kappagoda, 2009). Among all the tonics in traditional Chinese medicine (TCM), Herba *Cistanche* is widely accepted as a superior one and has even been given the name “Ginseng of the deserts.” In TCM, Herba *Cistanche* is frequently prescribed to treat chronic renal disease, impotence, female infertility, morbid leucorrhea, profuse metrorrhagia, and senile constipation (Zhang et al., 2005). In 2000 and 2005, respectively, *C. tubulosa*, and *C. deserticola* were indexed in the Chinese Pharmacopeia (Pharmacopoeia, 2000) (**Figure 1**). *C. tubulosa* is offered as an alternative for *C. deserticola* because of its similar chemical constituents and pharmacological activities and its abundance (Pharmacopoeia, 2005). Other species of this genus, e.g., *C. salsa* and *C. sinensis*, are also used as alternatives in some areas. *Cistanche* is considered a new cultivated plant in several regions in northwestern China, where the rainfall is low and soil desertification is severe.

**FIGURE 1:**
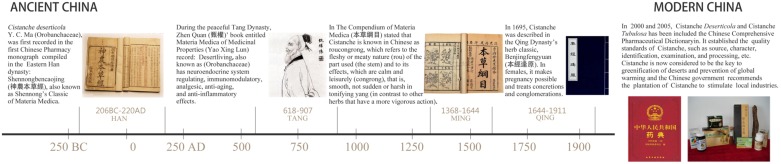
**Timeline of Herba *Cistanche* records in the traditional Chinese herb classic texts**. The uses of Herba *Cistanche* have evolved over nearly 2,000 years. Herba *Cistanche* was described in the oldest surviving herb classic, Shennong Bencao Jing (approximately 100 A.D.). Since then, it has been described in many famous traditional Chinese herb classics spanning different dynasties, including Yaoxinglun, The Compendium of Materia Medica (Bencaogangmu), and Benjingfengyuan. It was the most frequently prescribed drug against chronic renal disease in China for successive dynasties. In 2000 and 2005, *C. deserticola* was reordered in the Chinese Pharmacopoeia (2000), and *C. tubulosa* was added to the Chinese Pharmacopoeia (2005) as an alternative. Products manufactured in China that are made from Herba *Cistanche* (e.g., its extracts) or that include the herb (e.g., prepared formulas) are not restricted.

Since the 1980s, researchers have been interested in Herba *Cistanche*. A chemical analysis of Herba *Cistanche* revealed that essential oils, phenylethanoid glycosides (PhGs), iridoids, lignans, alditols, oligosaccharides, cistanosides, and polysaccharides were the main constituents (Jin and Zhang, 1994). Herba *Cistanche* extracts are pharmacologically active, with a range of functions that include improving chronic renal disease and senile constipation, increasing learning/memorizing ability, treating Alzheimer’s disease (AD), and improving immunity (Snytnikova et al., 2012; Zhang et al., 2012; Guo et al., 2013; Li et al., 2013a; Nan et al., 2013). Most pharmaceutical companies are hoping to find the next ‘miracle’ drug, such as artemisinin (qinghaosu), an antimalarial drug that is extracted from the medicinal plant sweet wormwood and has saved millions of lives. The other classic example of a modern medicine that originated in TCM is arsenic trioxide, which was approved by the US Food and Drug Administration (FDA) to treat leukemia in 2000. The present overview focuses on the progress of the study of chemical constituents of Herba *Cistanche* and some of its relevant pharmacological activities.

## Chemical Constituents

### Volatile Compounds

In essential oil of the Herba *Cistanche*, alkanes, alcohols, aldehydes, and heterocyclics were detected, and palmitic acid, linoleic acid, 14-methylpentadecanoate, ethyl palmitate, and 2,5,6-trimethyloctane were identified. The total oils of *C. tubulosa* mainly consist of palmitic acid and linoleic acid, while the essential oil of *C. salsa* includes alkanes, alcohols, aldehydes, and some heterocyclic compounds (Jiang and Tu, 2009). The volatile compounds of Herba *Cistanche*, like the essential oils, commonly can be extracted by steam distillation or lipophilic organic solvent. By extracting *C. deserticola* with petroleum ether and then analyzing the sample on a GC-MS, 25 volatile compounds were identified, and the 3 richest constituents in the petroleum ether extract are methyl 14-methylpentadecanoate (13.61%), ethyl palmitate (12.39%), and 2,5,6-trimethyloctane (7.60%) (Jiang and Tu, 2009).

### Non-Volatile Compounds

Among the non-volatile compounds of Herba *Cistanche*, more than 100 have currently been isolated and identified. These compounds are mostly PhGs, iridoids, lignans, alditols, oligosaccharides, and polysaccharides. As an important class of the compounds that constitute Herba *Cistanche*, PhGs have been well studied (**Table 1**). To date, 34 PhG compounds have been successfully isolated from Herba *Cistanche*, including 22 disaccharide glycosides, 10 trisaccharide glycosides, and 2 monosaccharide glycosides. The empirical structural features of PhGs are as follows. (1) For disaccharide glycosides, the sugar moiety consists of glucose and rhamnose connected by a Glc (3 → 1) Rha linkage; the glucose commonly links directly to an aglycone, and a coumaroyl or caffeoyl is usually located at the C4 or C6 position. (2) For trisaccharide glycosides, there is another glucose or rhamnose at the C6 position of the inside glucose. Recently, Li et al. (2015) completed the first deep transcriptome sequencing of the fleshy stem of *C*. *deserticola* by RNA-seq and identified some key enzyme genes and pathways that are involved in the biosynthesis of lignin and PhGs, which provides valuable information for this medical plant.

**Table 1:** Pharmaceutical effects of the active components from Herba *Cistanche.*

Active ingredient	Functions	Related diseases	Pathways	Research model	Reference
Phenylethanoid glycosides (PhGs)	Antioxidation, neuroprotection	PD	Preventing MPP^+^-induced apoptosis	Rat cerebella granule neurons (CGNs)	Tian and Pu, 2005
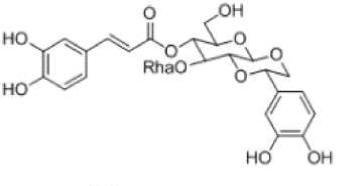	Neuroprotection, enhancing sexual function	–	–	Mice	Sato et al., 1985
	Enhancing immunity	–	Increasing T lymphocyte transformation	Mouse T cells	Shen et al., 1995
	Antioxidation, hepatoprotection	–	XOD inhibition	Rat liver microsome	Xiong et al., 1996, 1998
	Neuroprotection	AD	–	Clinical trial	Guo et al., 2013
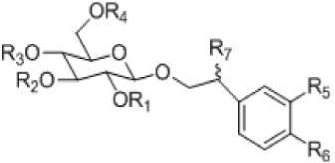	Antioxidation	–	Repairing OH⋅-induced DNA damage	Spin trapping	Wang et al., 2001
	Neuroprotection	AD	Increasing bax expression	Aβ ^25-35^-induced AD PC12 model	Luo et al., 2010
Echinacoside	Neuroprotection	PD	–	Mouse MPTP model	Geng et al., 2007
	Neuroprotection	–	Inhibition of caspase-3 activity, increase Bcl2 expression	SHSY5Y (human neuroblastoma) cells	He et al., 2009b
	Endothelium-dependent relaxation	Vascular diseases, sexual dysfunction	NO-cGMP pathways	Rat thoracic aortic rings	He et al., 2009b
Acteoside	Neuroprotection	–	Preventing MPP^+^-induced apoptosis	Rat CGNs	Pu et al., 2003
	Anti-allergy	Type I allergy	Ca/NFAT and JNK MAPK	KU812 (human basophilic) cells	Motojima et al., 2013
Tubuloside B	Neuroprotection	Neurodegenerative diseases	Preventing MPP^+^-induced apoptosis	Rat PC12 neuronal cells	Sheng et al., 2002
	Neuroprotection	Neurodegenerative diseases	Maintain mitochondria function, decrease concentration of free intracellular calcium and inhibit caspase-3 activity	SHSY5Y (human neuroblastoma) cells	Deng et al., 2004a

*PD, parkinson’s disease; AD, Alzheimer’s disease; XOD, xanthine/xanthine oxidase; MPP^+^, 1-methyl-4-phenylpyridinium*.

For the other non-volatile compounds of Herba *Cistanche*, 3 iridoid aglycones and 14 iridoid glycosides have currently been isolated from *cistanche* species (Xie et al., 2006); 1 and 5 lignan glycosides have been isolated from *C. deserticola* and *C. tubulosa*, respectively. Only 2 alkaloids, betaine and N,N-dimethyl glycine methyl ester, have been isolated from Herba *Cistanche* (Jiang and Tu, 2009). Small amounts of other compounds, such as phenolic glycosides, sterols or their glycosides, fatty acids, amino acids, and trace elements, are also present in Herba *Cistanche* (Snytnikova et al., 2012).

## Pharmacological Activities

Based on the neuroprotection, immune-enhancement, and sexual health properties of Herba *Cistanche*, we had a discussion in the paper (Supplementary Figure S1).

### Improvement of Brain Function

When considering learning and memory, three levels of mechanisms are involved: (1) the ability to acquire memory, i.e., learning ability; (2) the ability to store memory, i.e., consolidation; and (3) the ability to recall memorized information (Choi et al., 2011). The *C. tubulosa* extract was confirmed to significantly improve these mechanisms by preventing brain neuron apoptosis through the expression of apoptosis-related factors and neurotrophic factors in MES23.5 cells (Lin et al., 2013). *C. tubulosa* extract, containing rich echinacoside, and acteoside, can alleviate the cognitive dysfunction caused by Aβ^1-42^ through blocking amyloid deposition, reversing cholinergic, and hippocampal dopaminergic neuronal function in AD-like rat model (Wu et al., 2014). One of the PhGs, echinacoside, is typically known as the main phenolic component in the roots of *Echinacea angustifolia*, which is widely used in Europe and North America for its immunoregulation properties. A recent study determined that echinacoside can rescue human fibroblasts (SHSY5Y) from TNFα-induced apoptosis. The results indicated that echinacoside protects the damaged fibroblasts by regulating the reactive oxygen species level in fibroblasts and the activation of caspase-3 (Zhao et al., 2010). *In vitro* and *in vivo* experiments have confirmed that individual PhGs can inhibit the apoptosis of neuronal cells induced by various chemicals (Tian and Pu, 2005; Geng et al., 2007). Therefore, the individual PhGs could be attractive candidates against some typical neurodegenerative disorders, such as dementia or Parkinson’s disease (PD). Moreover, an open-label, non-placebo-controlled study on *C. tubulosa* glycoside capsules (Memoregain^®^) demonstrates that the drug has a potential to be a possible treatment option for mild to moderate AD, and all adverse reactions were mild. (Guo et al., 2013).

Similarly, the *C. salsa* extract can accelerate the proliferation of fibroblasts and promote the production of neurons by accelerating the growth of the neurites. It also has some properties as follows: prevent damage caused by cerebral ischemia-reperfusion; protect against apoptosis of the CA1 region of hippocampus (Wang et al., 2004); and increase the amount of neurotransmitters, such as dopamine (DA), noradrenaline (NA), and serotonin (5-HT), in the rat brain (Chen et al., 2007; Choi et al., 2011; Zhong et al., 2012). The acteoside-rich fraction of *C. salsa* extract can inhibit reactive oxygen species, prevent DNA damage, enhance superoxide dismutase (SOD) activity and prevent lipid peroxidation (Lin et al., 2002; Deng et al., 2004b; He et al., 2009a). Because acteoside has an extremely strong antioxidative effect that is 15 times stronger than resveratrol and five times stronger than vitamin C (Chiou et al., 2004).

### Aphrodisiac Effect

A recent study has demonstrated that an ethanol extract of *C. tubulosa* could increase the sex hormone levels by inducing testicular steroidogenic enzymes (e.g., CYP11A1, CYP17A1, CYP3A4) (Wang et al., 2015). Besides, the gene expression of 3β-hydroxysteroid dehydrogenase (3β-HSD), which is responsible for the synthesis of testosterone, 5α-reductase-2 and aldo-keto reductase (enzymes that are responsible for the synthesis of dihydrotestosterone), can be induced by the *C. tubulosa* extract, suggesting the positive effect of *C. tubulosa* extract on male hormone production (Shimoda et al., 2009). The acteoside, which was distilled from *C. tubulosa*, significantly shortened the latent period of penis erection (*p* < 0.01), increased the number of germ cells (*p* < 0.01), and improved pathological changes in the testes (Ma et al., 2009). Echinacoside, which is another newly identified PhG and is typically known as the main component of echinacea, is abundant in *C. tubulosa* and possesses vaso-relaxing activity (Yoshikawa et al., 2006). The aphrodisiac mechanisms of *C. tubulosa* might involve the NO-cGMP signal transduction pathway, with increasing cGMP levels in the corpus cavernosum smooth muscle (He et al., 2009b). Pan and Min (2004) indicated that the combined use of Herba *Cistanche* extract could prevent adrenal cortical atrophy, which is typically caused by using corticosteroids only. Moreover, *C. deserticola* extract could reverse the reproductive toxicity in mice induced by hydroxyurea (Gu et al., 2013) and glycoside of Leigongteng (*Radix et Rhizoma Tripterygii*) (Li et al., 2014).

According to TCM, Yang-Qi Kidney-Yang Deficiency Syndrome (KDS-Yang) is caused by insufficient “Yang-Qi” in the kidney. Briefly, Yang-Qi is a TCM term and likely indicates mitochondrion-driven biological activities of the human body in the view of biomedical research (Leong et al., 2015). In H9c2 cardiomyocytes, Herba *Cistanche* was proven to enhance the mitochondrial respiration and glutathione antioxidant status (Wong and Ko, 2013). Yang-Qi deficiencies in TCM resemble those of chronic fatigue syndrome in Western medicine. KDS-Yang symptoms include soreness and weakness of the waist and knees, cold chills, deafness, and tinnitus. Modern studies showed that damage and functional disorders of the hypothalamic-pituitary-target gland axis, including the adrenal gland, thyroid, and gonad, are the main pathological mechanisms of KDS-Yang (Zhao et al., 2013). Gong et al. (2008) investigated the intervention effect of *C. deserticola* (decoction, 10 g/kg⋅d) in hydrocortisone-induced KDS-Yang model rats and found that *C. deserticola* extract could increase body weight, autonomic activity, and swimming time while decreasing post-exercise blood lactic acid (LAC) and blood urea nitrogen (BUN). As described in the Compendium of Materia Medica, Herba *Cistanche* is mild and would not be extremely abrupt or harsh when treating KDS-Yang. Interestingly, KDS-Yang also leads to low and disordered immune function, and thus, there is a close relationship between treating KDS-Yang and improving immune function (Yim and Ko, 2002).

### Immune-Boosting Effect

Besides its traditional use, the daily consumption of Herba *Cistanche* is believed to the key of the people with longevity in some region of China and Japan where known for longevity and oasis. Studies shown that both *C. salsa* extract and *C. tubulosa* extract can activate lymphoid cells and increase the killed rate of cancer cells (Maruyama et al., 2009). *C. deserticola* extract can activate the phagocytic function of macrophages in mice and enhance body immunity (Li et al., 2009). Carbohydrates account for a high proportion of the dry mass of Herba *Cistanche*. The polysaccharides of C. *deserticola* are closely related to the immunity enhancing and anti-cancer functions (Xu et al., 2011). Galactitol is one of the monosaccharides in Herba *Cistanche* with laxative activity (Baishun et al., 2003). Individual compounds such as oligosaccharides present an excellent effect on the spleen activity of mice, increase the phagocytotic activity of macrophages and stimulate the proliferation of antibody-producing cells (Maruyama et al., 2008). Acteoside (10 or 50 mg/kg subcutaneously) significantly inhibits hepatic apoptosis, hepatitis and lethality in mice with hepatic apoptosis and liver failure were induced by D-galactosamine (DGalN) and lipopolysaccharide (LPS) (Xiong et al., 1999). Echinacoside-enriched extract of *C. tubulosa* is effective in preventing dextran sulphate sodium (DSS)-induced colitis in mice (Jia et al., 2014). Recently, Zhang et al. (2014) discovered that *C. deserticola* extract could antagonize immune-related senescence and extend the lifespan in SAM-P8 mice. In Zhang’s et al. (2014) study, dietary supplementation with C. *deserticola* extract can decrease the level of peripheral memory T cells and enhance levels of naive T cells. Additionally, Herba *Cistanche* aqueous extract was proven to prevent bone loss caused by ovarian hormone deficiency through regulating some bone metabolism related genes (e.g., Smad1, Smad5, TGF-b1, and TIEG1) (Liang et al., 2011, 2013), and amonoterpene from *C. salsa* has been identified as an anti-osteoporotic compound (Yamaguchi et al., 1999). Herba *Cistanche* helps people better understand the synergistic effect mechanisms of the effective components in TCM.

## Conclusion

Herba *Cistanche* has been commonly used traditionally for enhancing immunity, sexual health, antioxidation, and neuroprotection, and tonic. Various Herba *Cistanche* products and its derivative are widely used in modern China. The uses of this drug have evolved over nearly 2,000 years, and the demand for Herba *Cistanche* has grown rapidly in recent years. After a long-term use, those compounds with proven pharmacological activity, such as acteoside, and echinacoside, deserve more in-depth study before they truly could improve patient quality of life. With further study, this important TCM is believed to have profound prospects.

## Author Contributions

HL drafted the table. LG and JG designed the figure. ZL and CT drafted and revised the manuscript.

## Conflict of Interest Statement

The authors declare that the research was conducted in the absence of any commercial or financial relationships that could be construed as a potential conflict of interest.

## Supplementary Material

FIGURE S1 **Main biological functions and mechanisms of the active components of Herba *Cistanche***. The active ingredients isolated from Herba *Cistanche* have three main biological functions: improvement of brain function, immune-boosting effect, and nourishing aphrodisiac effect. Herba *Cistanche* extracts are mixtures of multiple active compounds, and a typical traditional Chinese medicine (TCM) recognizes multiple biological targets. Phenylethanoid glycosides (PhGs) have neuroprotective properties in neurodegenerative disease based on their free radical scavenging ability, anti-apoptosis activity, anti-inflammation activity, the increasing amount of brain neurotransmitters and enhanced SOD activity. A modern use of Herba *Cistanche* in Chinese herbalism is to treat KDS-Yang, which contributes to fertility problems (including impotence and female infertility) because Herba *Cistanche* is different from other herbs that often have a more vigorous action. Herba *Cistanche* regulates the hypothalamic-pituitary-gonad (HPG) and hypothalamic-pituitary-adrenal (PHA) axes, which may induce a smooth and balanced sexual vitality effect. Moreover, phenylethanoid oligoglycosides and acylated oligosaccharides from Herba *Cistanche* have vasorelaxant activity via the NO-cGMP pathway. Based on clinical and experimental data, activation of the HPA axis increases plasma cytokines and acute phase protein concentrations. Thus, it can increase phytohemagglutinin-stimulated lymphocyte proliferation and enhance antibody production in response to T-dependent antigens in spleen cells.
