# Supplementary figures and images for: Herba Cistanche (Rou Cong-Rong): One of the Best Pharmaceutical Gifts of Traditional Chinese Medicine
Source: Front Pharmacol. 2016 Mar 1;7:41. doi: 10.3389/fphar.2016.00041 (PMC4771771; doi:10.3389/fphar.2016.00041)

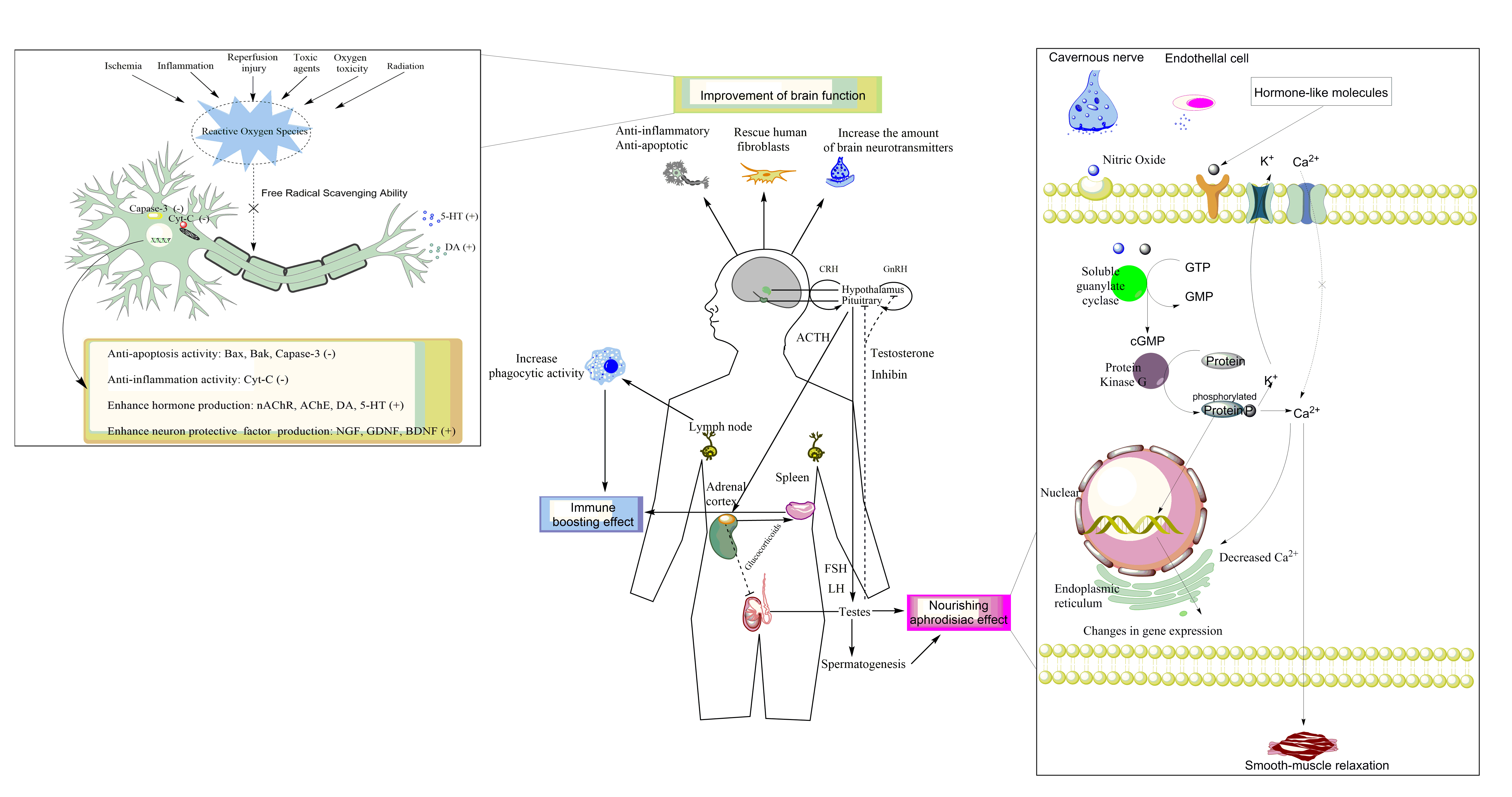

Supplement: FIGURE S1 — Main biological functions and mechanisms of the active components of Herba Cistanche. The active ingredients isolated from Herba Cistanche have three main biological functions: improvement of brain function, immune-boosting effect, and nourishing aphrodisiac effect. Herba Cistanche extracts are mixtures of multiple active compounds, and a typical traditional Chinese medicine (TCM) recognizes multiple biological targets. Phenylethanoid glycosides (PhGs) have neuroprotective properties in neurodegenerative disease based on their free radical scavenging ability, anti-apoptosis activity, anti-inflammation activity, the increasing amount of brain neurotransmitters and enhanced SOD activity. A modern use of Herba Cistanche in Chinese herbalism is to treat KDS-Yang, which contributes to fertility problems (including impotence and female infertility) because Herba Cistanche is different from other herbs that often have a more vigorous action. Herba Cistanche regulates the hypothalamic-pituitary-gonad (HPG) and hypothalamic-pituitary-adrenal (PHA) axes, which may induce a smooth and balanced sexual vitality effect. Moreover, phenylethanoid oligoglycosides and acylated oligosaccharides from Herba Cistanche have vasorelaxant activity via the NO-cGMP pathway. Based on clinical and experimental data, activation of the HPA axis increases plasma cytokines and acute phase protein concentrations. Thus, it can increase phytohemagglutinin-stimulated lymphocyte proliferation and enhance antibody production in response to T-dependent antigens in spleen cells. [file Image_1.JPEG]
